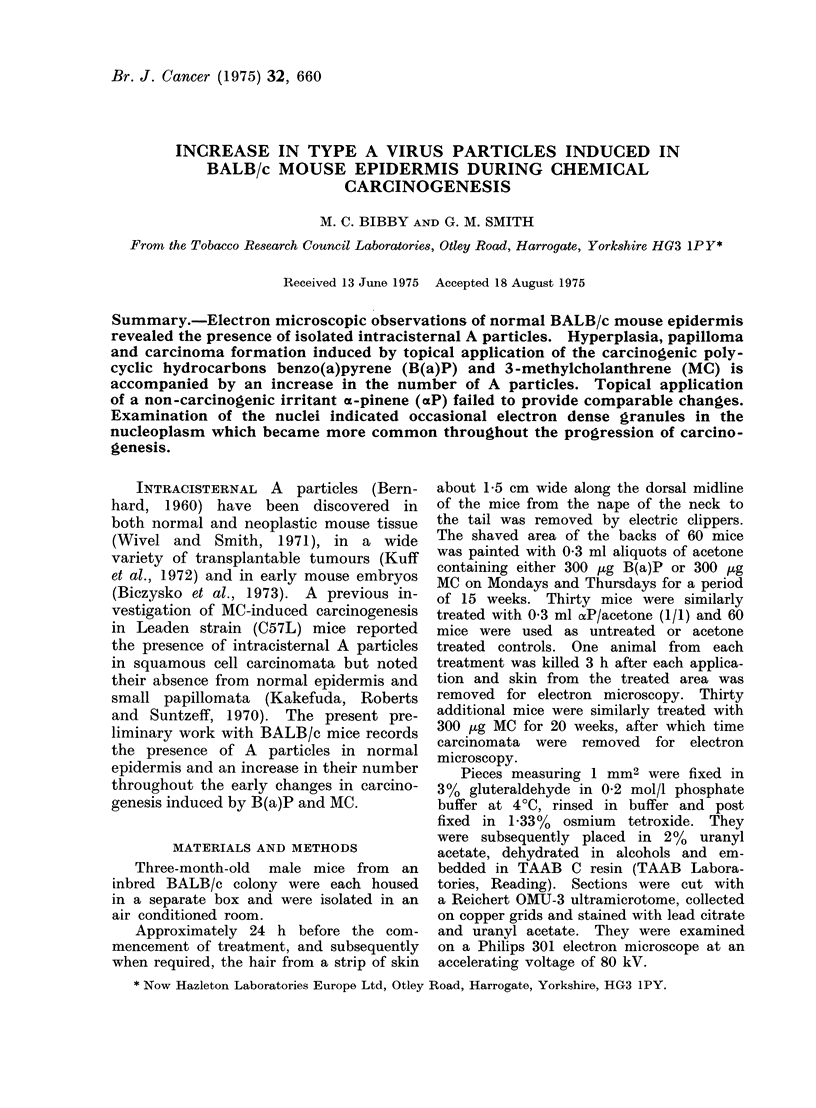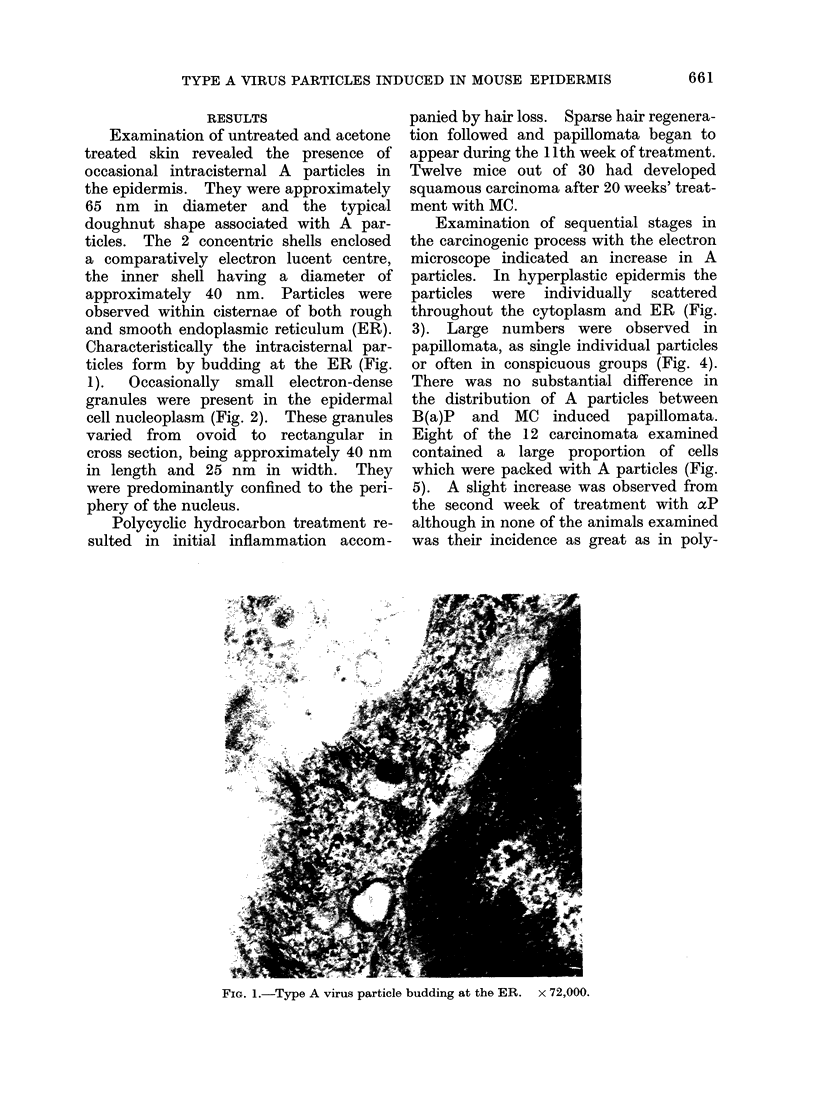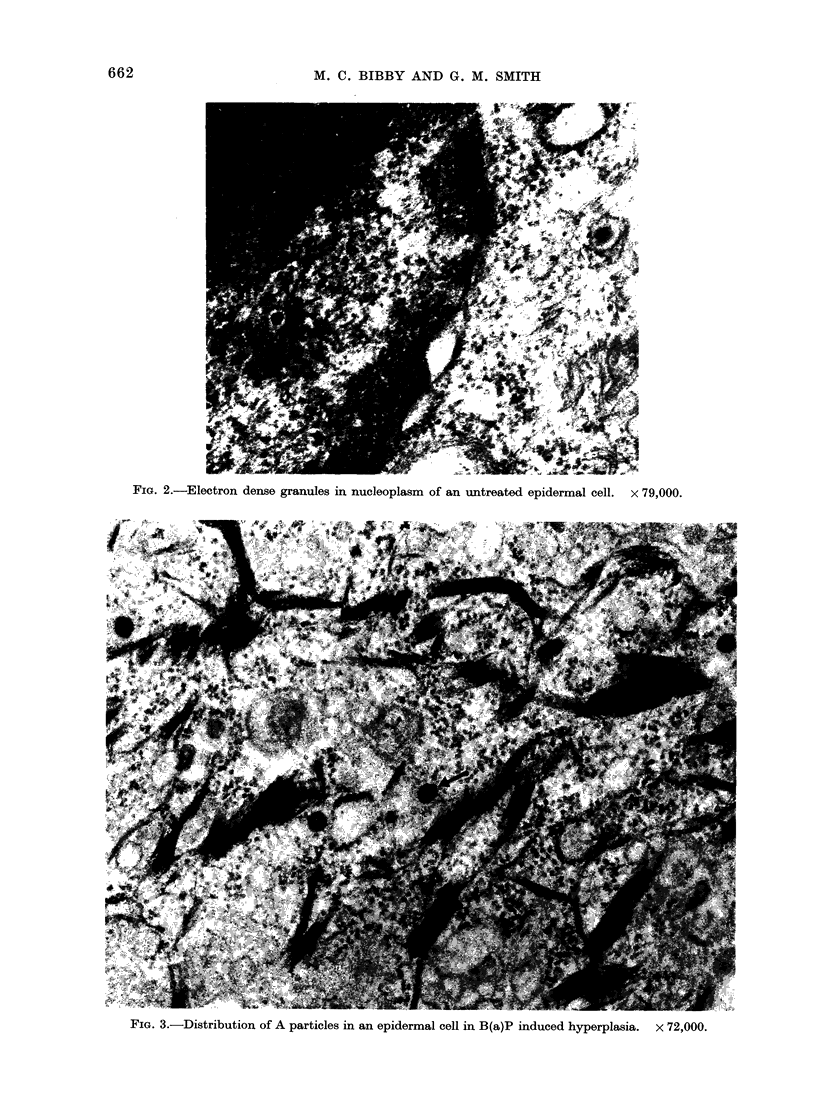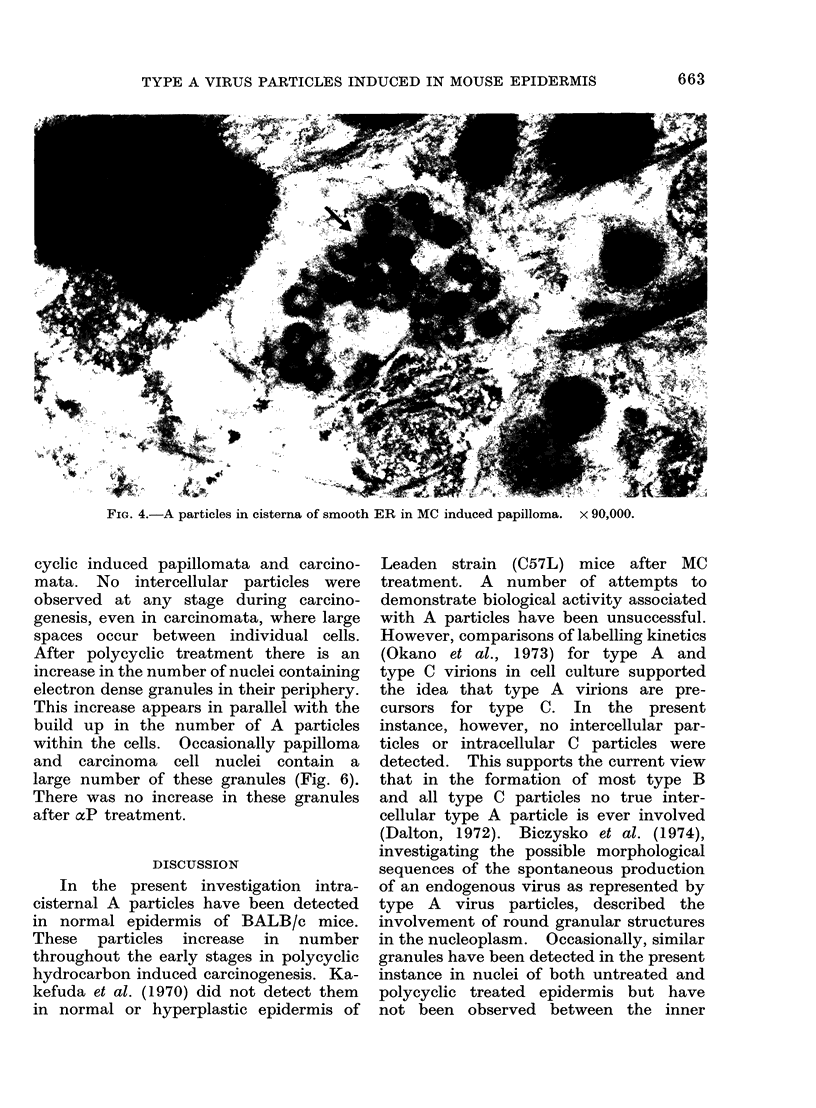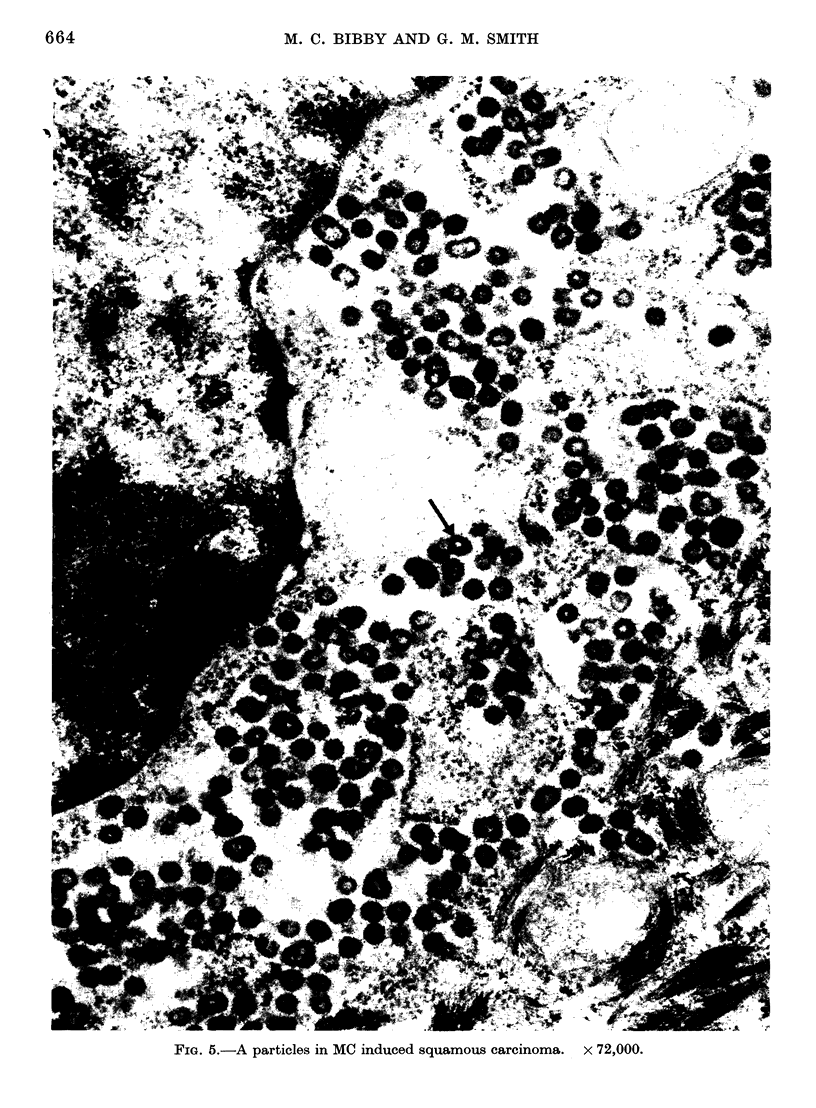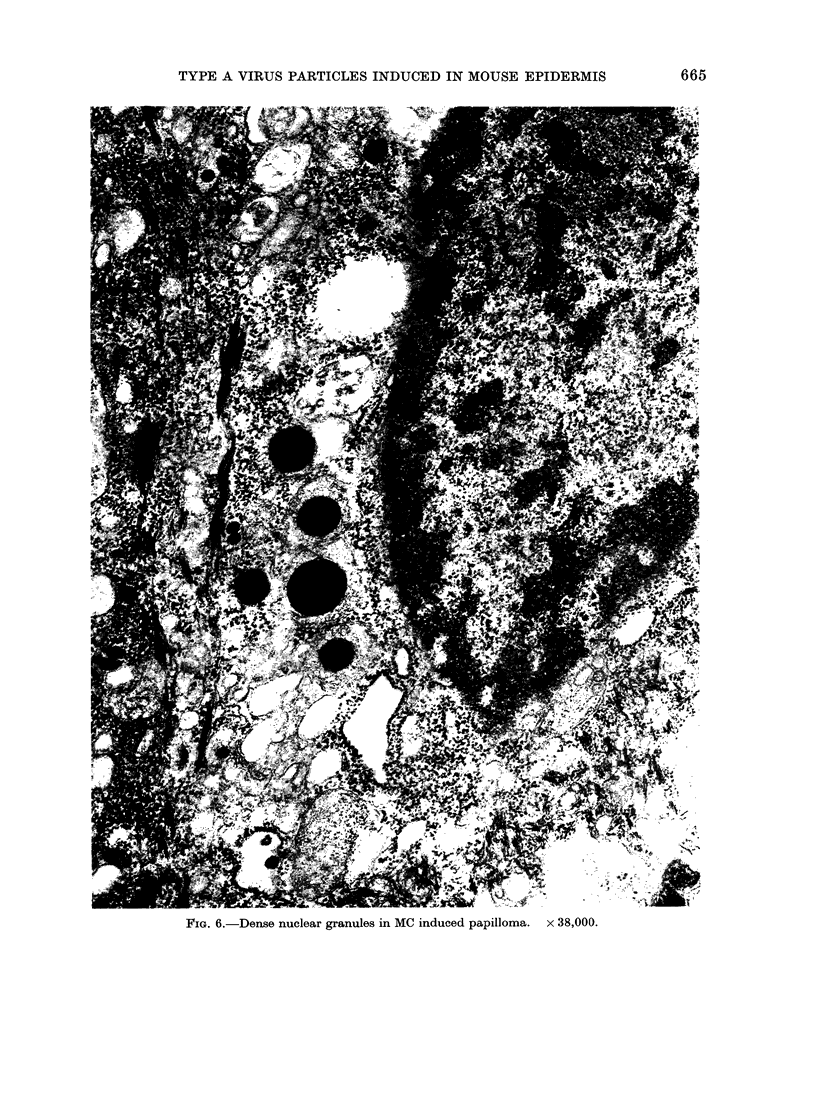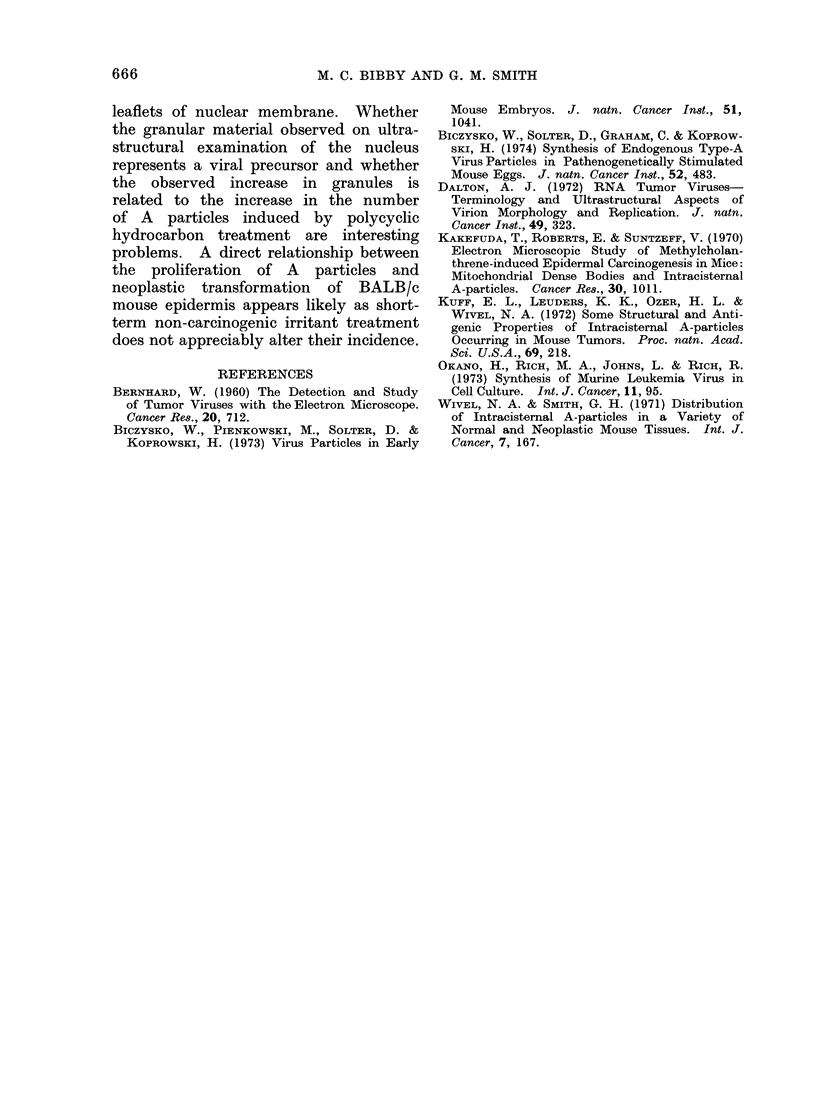# Increase in type A virus particles induced in BALB/c mouse epidermis during chemical carcinogenesis.

**DOI:** 10.1038/bjc.1975.276

**Published:** 1975-12

**Authors:** M. C. Bibby, G. M. Smith

## Abstract

**Images:**


*Br. J. Cancer* (1975) **32**, 660

### INCREASE IN TYPE A VIRUS PARTICLES INDUCED IN BALB/c MOUSE EPIDERMIS DURING CHEMICAL CARCINOGENESIS

M. C. BIBBY AND G. M. SMITH

*From the Tobacco Research Council Laboratories, Otley Road, Harrogate, Yorkshire HG3 1PY**

Received 13 June 1975 Accepted 18 August 1975

**Summary.—Electron microscopic observations of normal BALB/c mouse epidermis revealed the presence of isolated intracisternal A particles. Hyperplasia, papilloma and carcinoma formation induced by topical application of the carcinogenic polycyclic hydrocarbons benzo(a)pyrene (B(a)P) and 3-methylcholanthrene (MC) is accompanied by an increase in the number of A particles. Topical application of a non-carcinogenic irritant α-pinene (αP) failed to provide comparable changes. Examination of the nuclei indicated occasional electron dense granules in the nucleoplasm which became more common throughout the progression of carcinogenesis.**

INTRACISTERNAL A particles (Bernhard, 1960) have been discovered in both normal and neoplastic mouse tissue (Wivel and Smith, 1971), in a wide variety of transplantable tumours (Kuff *et al.*, 1972) and in early mouse embryos (Biczysko *et al.*, 1973). A previous investigation of MC-induced carcinogenesis in Leaden strain (C57L) mice reported the presence of intracisternal A particles in squamous cell carcinomata but noted their absence from normal epidermis and small papillomata (Kakefuda, Roberts and Suntzeff, 1970). The present preliminary work with BALB/c mice records the presence of A particles in normal epidermis and an increase in their number throughout the early changes in carcinogenesis induced by B(a)P and MC.

### MATERIALS AND METHODS

Three-month-old male mice from an inbred BALB/c colony were each housed in a separate box and were isolated in an air conditioned room.

Approximately 24 h before the commencement of treatment, and subsequently when required, the hair from a strip of skin about 1·5 cm wide along the dorsal midline of the mice from the nape of the neck to the tail was removed by electric clippers. The shaved area of the backs of 60 mice was painted with 0·3 ml aliquots of acetone containing either 300 μg B(a)P or 300 μg MC on Mondays and Thursdays for a period of 15 weeks. Thirty mice were similarly treated with 0·3 ml αP/acetone (1/1) and 60 mice were used as untreated or acetone treated controls. One animal from each treatment was killed 3 h after each application and skin from the treated area was removed for electron microscopy. Thirty additional mice were similarly treated with 300 μg MC for 20 weeks, after which time carcinomata were removed for electron microscopy.

Pieces measuring 1 $mm^2$ were fixed in 3% gluteraldehyde in 0·2 mol/l phosphate buffer at 4°C, rinsed in buffer and post fixed in 1·33% osmium tetroxide. They were subsequently placed in 2% uranyl acetate, dehydrated in alcohols and embedded in TAAB C resin (TAAB Laboratories, Reading). Sections were cut with a Reichert OMU-3 ultramicrotome, collected on copper grids and stained with lead citrate and uranyl acetate. They were examined on a Philips 301 electron microscope at an accelerating voltage of 80 kV.

* Now Hazleton Laboratories Europe Ltd, Otley Road, Harrogate, Yorkshire, HG3 1PY.

### RESULTS

Examination of untreated and acetone treated skin revealed the presence of occasional intracisternal A particles in the epidermis. They were approximately 65 nm in diameter and the typical doughnut shape associated with A particles. The 2 concentric shells enclosed a comparatively electron lucent centre, the inner shell having a diameter of approximately 40 nm. Particles were observed within cisternae of both rough and smooth endoplasmic reticulum (ER). Characteristically the intracisternal particles form by budding at the ER (Fig. 1). Occasionally small electron-dense granules were present in the epidermal cell nucleoplasm (Fig. 2). These granules varied from ovoid to rectangular in cross section, being approximately 40 nm in length and 25 nm in width. They were predominantly confined to the periphery of the nucleus.

Polycyclic hydrocarbon treatment resulted in initial inflammation accompanied by hair loss. Sparse hair regeneration followed and papillomata began to appear during the 11th week of treatment. Twelve mice out of 30 had developed squamous carcinoma after 20 weeks' treatment with MC.

Examination of sequential stages in the carcinogenic process with the electron microscope indicated an increase in A particles. In hyperplastic epidermis the particles were individually scattered throughout the cytoplasm and ER (Fig. 3). Large numbers were observed in papillomata, as single individual particles or often in conspicuous groups (Fig. 4). There was no substantial difference in the distribution of A particles between B(a)P and MC induced papillomata. Eight of the 12 carcinomata examined contained a large proportion of cells which were packed with A particles (Fig. 5). A slight increase was observed from the second week of treatment with $\alpha$P although in none of the animals examined was their incidence as great as in poly-

FIG. 1.—Type A virus particle budding at the ER. ×72,000.

FIG. 2.—Electron dense granules in nucleoplasm of an untreated epidermal cell. × 79,000.

FIG. 3.—Distribution of A particles in an epidermal cell in B(a)P induced hyperplasia. × 72,000.

FIG. 4.—A particles in cisterna of smooth ER in MC induced papilloma. ×90,000.

cyclic induced papillomata and carcinomata. No intercellular particles were observed at any stage during carcinogenesis, even in carcinomata, where large spaces occur between individual cells. After polycyclic treatment there is an increase in the number of nuclei containing electron dense granules in their periphery. This increase appears in parallel with the build up in the number of A particles within the cells. Occasionally papilloma and carcinoma cell nuclei contain a large number of these granules (Fig. 6). There was no increase in these granules after αP treatment.

### DISCUSSION

In the present investigation intracisternal A particles have been detected in normal epidermis of BALB/c mice. These particles increase in number throughout the early stages in polycyclic hydrocarbon induced carcinogenesis. Kakefuda *et al.* (1970) did not detect them in normal or hyperplastic epidermis of Leaden strain (C57L) mice after MC treatment. A number of attempts to demonstrate biological activity associated with A particles have been unsuccessful. However, comparisons of labelling kinetics (Okano *et al.*, 1973) for type A and type C virions in cell culture supported the idea that type A virions are precursors for type C. In the present instance, however, no intercellular particles or intracellular C particles were detected. This supports the current view that in the formation of most type B and all type C particles no true intercellular type A particle is ever involved (Dalton, 1972). Biczysko *et al.* (1974), investigating the possible morphological sequences of the spontaneous production of an endogenous virus as represented by type A virus particles, described the involvement of round granular structures in the nucleoplasm. Occasionally, similar granules have been detected in the present instance in nuclei of both untreated and polycyclic treated epidermis but have not been observed between the inner

FIG. 5.—A particles in MC induced squamous carcinoma. ×72,000.

FIG. 6.—Dense nuclear granules in MC induced papilloma. ×38,000.

leaflets of nuclear membrane. Whether the granular material observed on ultrastructural examination of the nucleus represents a viral precursor and whether the observed increase in granules is related to the increase in the number of A particles induced by polycyclic hydrocarbon treatment are interesting problems. A direct relationship between the proliferation of A particles and neoplastic transformation of BALB/c mouse epidermis appears likely as short-term non-carcinogenic irritant treatment does not appreciably alter their incidence.

### REFERENCES

BERNHARD, W. (1960) The Detection and Study of Tumor Viruses with the Electron Microscope. *Cancer Res.*, **20**, 712.

BICZYSKO, W., PIENKOWSKI, M., SOLTER, D. & KOPROWSKI, H. (1973) Virus Particles in Early Mouse Embryos. *J. natn. Cancer Inst.*, **51**, 1041.

BICZYSKO, W., SOLTER, D., GRAHAM, C. & KOPROWSKI, H. (1974) Synthesis of Endogenous Type-A Virus Particles in Pathenogenetically Stimulated Mouse Eggs. *J. natn. Cancer Inst.*, **52**, 483.

DALTON, A. J. (1972) RNA Tumor Viruses—Terminology and Ultrastructural Aspects of Virion Morphology and Replication. *J. natn. Cancer Inst.*, **49**, 323.

KAKEFUDA, T., ROBERTS, E. & SUNTZEFF, V. (1970) Electron Microscopic Study of Methylcholanthrene-induced Epidermal Carcinogenesis in Mice: Mitochondrial Dense Bodies and Intracisternal A-particles. *Cancer Res.*, **30**, 1011.

KUFF, E. L., LEUDERS, K. K., OZER, H. L. & WIVEL, N. A. (1972) Some Structural and Antigenic Properties of Intracisternal A-particles Occurring in Mouse Tumors. *Proc. natn. Acad. Sci. U.S.A.*, **69**, 218.

OKANO, H., RICH, M. A., JOHNS, L. & RICH, R. (1973) Synthesis of Murine Leukemia Virus in Cell Culture. *Int. J. Cancer*, **11**, 95.

WIVEL, N. A. & SMITH, G. H. (1971) Distribution of Intracisternal A-particles in a Variety of Normal and Neoplastic Mouse Tissues. *Int. J. Cancer*, **7**, 167.